# An unexpected challenge: intra-abdominal neurofibromatosis during biliary reconstruction: a case report

**DOI:** 10.1097/MS9.0000000000005545

**Published:** 2026-08-25

**Authors:** Alireza Firouzfar, Pouria Abedini, Parsa Elyasi Bakhtiari, Behnam Sanei

**Affiliations:** a Department of General Surgery, School of Medicine, Isfahan University of Medical Sciences, Isfahan, Iran; b Student Research Committee, School of Medicine, Isfahan University of Medical Sciences, Isfahan, Iran

**Keywords:** choledocholithiasis, common bile duct, gall stones, neurofibromatosis 1, viscera

## Abstract

**Introduction and importance:**

Neurofibromatosis type 1 (NF1) is a genetic disorder characterized by peripheral nerve sheath tumors in the skin or nervous system. Its prevalence is 1 in 2600–3000 individuals, but intra-abdominal manifestations appear in only 5–25% of these patients, which makes this presentation a rare medical condition. The presence of intra-abdominal lesions can significantly complicate surgical procedures and render biliary reconstruction technically challenging.

**Case presentation:**

This case describes an uncommon presentation of visceral NF1 in a 75-year-old woman with cutaneous NF1 lesions, who was admitted to our hospital with right upper quadrant pain and jaundice. Preoperative imaging studies showed a dilated and tortuous common bile duct, filled with multiple stones. Because of prior unsuccessful endoscopic retrograde cholangiopancreatography procedures, an elective choledochotomy was performed. During surgery, widespread intra-abdominal neurofibromas were noted throughout the small intestine, posing significant difficulty in finding a suitable, tumor-free site for biliary-enteric anastomosis.

**Clinical discussion:**

Visceral involvement in NF1 is rare but can present major diagnostic and surgical challenges. Gastrointestinal neurofibromas may remain asymptomatic until they cause obstruction, bleeding, or jaundice. Surgical management demands special consideration when neurofibromas diffusely infiltrate the mesentery or bowel wall, as they complicate both exposure and reconstruction. The main challenge of this case was finding an area without neurofibroma lesions in the small intestine for anastomosis to prevent postoperative complications.

**Conclusion:**

This case highlights the importance of managing patients with gastrointestinal manifestations of NF1 who require abdominal surgical intervention.

## Introduction

Neurofibromatosis type 1 (NF1), or von Recklinghausen disease, is an autosomal dominant disorder characterized by peripheral nerve sheath tumors affecting the skin or nervous system^[^1^]^. The main clinical manifestations are cutaneous neurofibromas, café-au-lait spots, axillary and inguinal freckling, and hamartomas or Lisch nodules of the iris^[^2^]^. Diagnosis is based on these clinical features and symptoms^[^3^]^.

The prevalence of NF1 is approximately 1 in 2600–3000 individuals. However, neoplastic intra-abdominal manifestations may appear in only 5–25% of patients with NF1. The rarity of intra-abdominal lesions in NF1 and their nonspecific symptoms, compared with the cutaneous type, have made diagnosis and treatment challenging^[^3,4^]^. In previous studies, involvement of the liver, colon, and small intestine has been reported in patients with intra-abdominal lesions^[^5–7^]^. Non-specific symptoms are the main characteristic of gastrointestinal NF1, including abdominal pain, obstruction, and jaundice^[^8^]^.

HIGHLIGHTS

- Rare intra-abdominal NF1 complicates biliary reconstruction.
- Neurofibromas made the selection of a safe anastomosis site challenging.
- The case stresses careful planning in abdominal surgery for visceral NF1.

Because of the complexity of the situation that arose during surgery, this case is noteworthy. The major challenge of this procedure was locating a region of the intestine free of neurofibroma lesions to ensure an optimal anastomosis, reduce leakage, and prevent other potential complications.

## Case presentation

This case describes a 75-year-old Iranian female, a known case of von Recklinghausen disease (with café-au-lait spots and neurofibromatosis lesions on her body), who presented with right upper quadrant pain and jaundice. The patient did not report any symptoms of gastrointestinal neurofibromas, such as constipation, obstruction, or an abdominal mass. No past medical history of hypertension or diabetes mellitus was reported. She had undergone appendectomy and a hernia repair in recent years. The patient had undergone laparoscopic cholecystectomy 8 years previously, followed by three endoscopic retrograde cholangiopancreatography (ERCP) procedures due to common bile duct (CBD) stones, with the most recent occurring 5 years ago. Despite these interventions, she continued to experience abdominal pain, indicative of choledocholithiasis. The case has been written in accordance with the SCARE checklist^[^9^]^.

### Pre-operative

**Figure 1.**
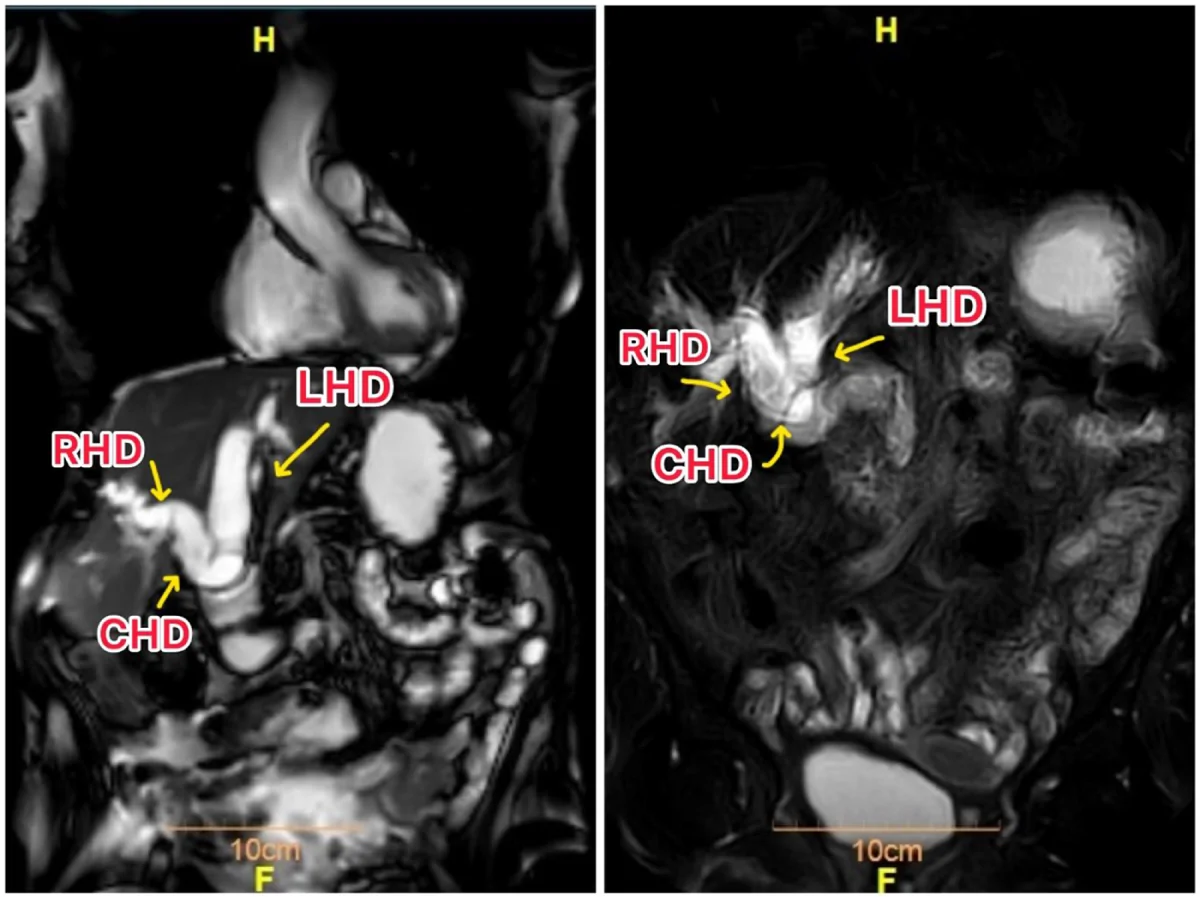
Preoperative imaging demonstrating marked dilation and tortuosity of the common hepatic duct (CHD), right hepatic duct (RHD), and left hepatic duct (LHD), with multiple intraductal stones. The LHD, RHD, and CHD are indicated by yellow arrows.

Imaging studies, such as sonography and magnetic resonance cholangiopancreatography, were performed as part of the preoperative evaluation for diagnostic purposes and to rule out other medical conditions, such as malignancy. These revealed a significantly dilated and tortuous CBD, with a maximum diameter of 30 mm that was filled with stones, as well as significant ectasia of the intrahepatic biliary ducts (Fig. 1).

Initial laboratory tests revealed a white blood cell count of 3820/µL, hemoglobin of 12.4 g/dL, a platelet count of 137 000/µL, an aspartate aminotransferase level of 84, an alanine transaminase level of 95, an alkaline phosphatase level of 839, a prothrombin time of 13.2 seconds, and a partial thromboplastin time of 39.6 seconds. Tumor markers were negative.

Given these findings and the previously unsuccessful ERCP procedures, the decision was made to perform an elective choledochotomy. Due to the presence of cholangitis, hydration and antibiotic therapy were commenced. Pre-operative evaluation was performed, and her ejection fraction was calculated to be 50–55% by echocardiography, as ordered.

### During operation

Appropriate exposure was achieved through a midline incision. Adhesion bands were observed during surgical exploration due to previous surgeries, necessitating meticulous enterolysis to improve access to the liver hilum. A choledochotomy was performed as soon as the surgical site was cleared.

The CBD was carefully explored and exposed, with great care taken to minimize injury to crucial tissues. Biliary sludge and numerous stones of various sizes (maximum size 30 mm) were removed, and the CBD was irrigated. After confirming the absence of stones, a Roux-en-Y choledochojejunostomy was performed on the patient.

**Figure 2.**
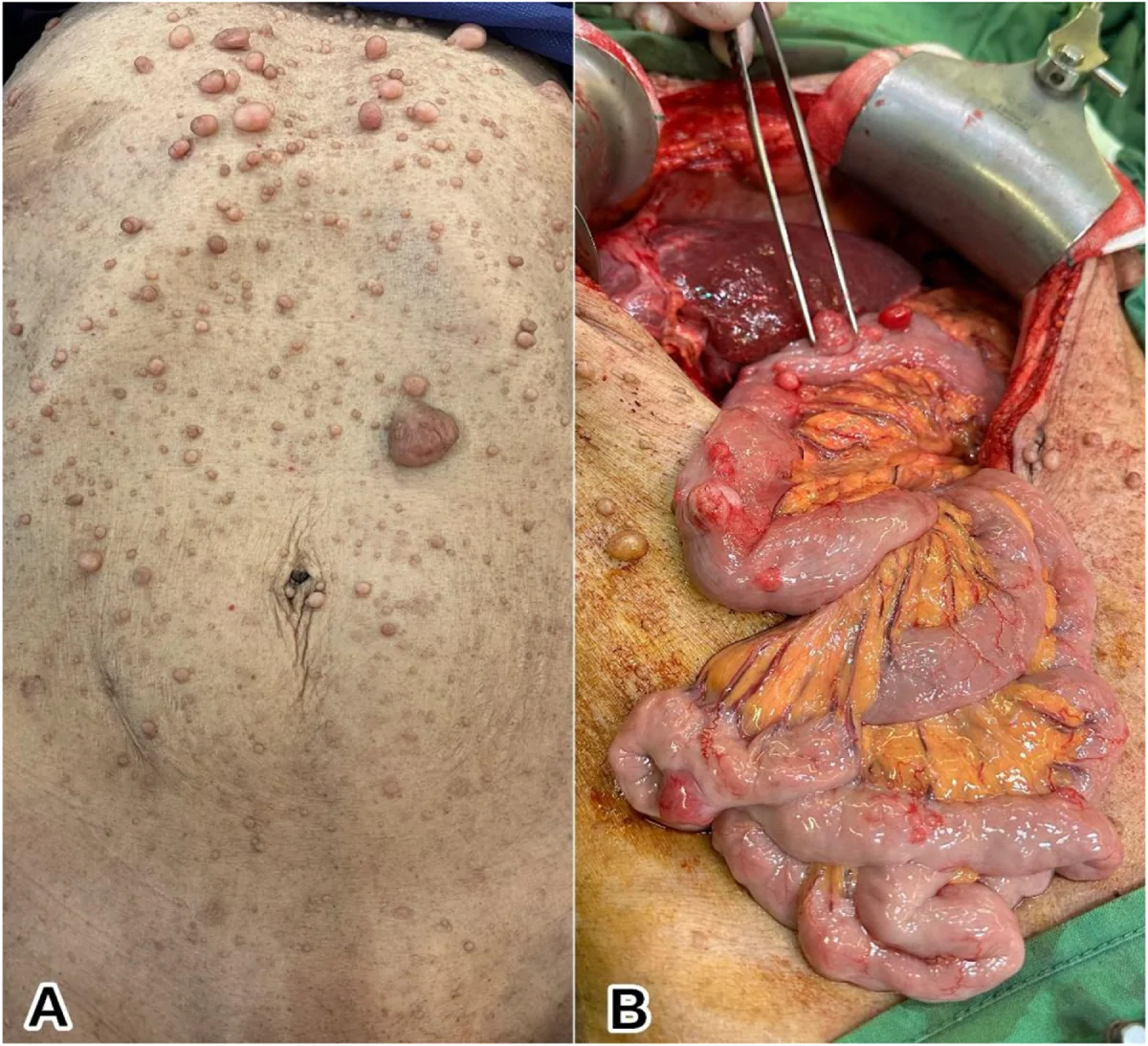
(A) Multiple cutaneous neurofibromas distributed over the patient’s trunk, consistent with neurofibromatosis type 1. (B) Intraoperative view demonstrating diffuse visceral neurofibromas involving the small intestine and liver. The widespread distribution of these lesions posed significant challenges in identifying a lesion-free segment suitable for biliary-enteric reconstruction.

The main challenge during surgery was identifying a suitable segment of the small intestine, devoid of lesions, to perform an anastomosis on the jejunal wall, as the procedure was complicated by widespread neurofibromas (Fig. 2).

In order to reduce the chance of difficulties and ensure a clear area for the anastomosis, the surgeon explored around the lesions. After dividing the jejunum with a linear cartridge stapler and bringing the Roux limb near the hepatic duct, a side-to-side anastomosis was created between the common hepatic duct and jejunal limb to make future ERCPs possible, if required.

In the next step, enteroenterostomy was performed. The presence of a neurofibroma limited this surgical step, as the surgeon had to find a clear site for optimal anastomosis to minimize postoperative complications such as leakage or obstruction.

### Post-operative management

The patient’s recovery was stable and uneventful, without any specific medical problems. She was also monitored for possible complications, such as bile leakage, but none were reported. A per oral (PO) regimen was started the following day, and on the fifth postoperative day, the patient was discharged in good general condition without complications.

At the 1-year follow-up, the patient remained completely asymptomatic, with no evidence of jaundice, abdominal pain, or biliary complications, and laboratory tests were within normal limits, confirming a satisfactory long-term outcome.

## Discussion

Visceral involvement in NF1 is uncommon and remains a diagnostically challenging manifestation because patients are often asymptomatic or present with nonspecific gastrointestinal symptoms^[^4^]^. Consequently, most cases are diagnosed incidentally during imaging or abdominal surgery performed for unrelated conditions.

Ait Ali *et al* reported a patient with intestinal obstruction and a solitary neurofibroma of the small bowel, who did not experience any clinical symptoms of gastrointestinal neurofibroma, similar to our case^[^5^]^.

A recent case report described the incidental intraoperative discovery of diffuse mesenteric neurofibromatosis during a laparoscopic appendectomy in a 17-year-old patient with NF1^[^10^]^. As in our case, the gastrointestinal involvement was asymptomatic and was unexpectedly identified during surgery for an unrelated abdominal condition. However, unlike that case, in which the mesenteric lesions did not alter the surgical procedure, our case presented a unique reconstructive challenge.

Al-Harake *et al* described a case of a patient with peptic ulcer disease and hiatal hernia who presented with epigastric abdominal pain. The patient was diagnosed with severe gastritis. Distention of the stomach, duodenum, jejunum, and ileum caused by intestinal intussusception due to an ileal tumor was obvious on CT imaging. Final investigations revealed an isolated intestinal neurofibroma in the ileum with no associated syndromes^[^11^]^.

In another study, Fujisawa *et al* reported on a 26-year-old man who underwent cholecystectomy. During the operation and imaging studies, he was diagnosed with intra-abdominal plexiform neurofibromatosis, including periportal, mesenteric, and gastrointestinal tract involvement^[^12^]^.

Another relevant report described a 32-year-old man with NF1 in whom diffuse intestinal ganglioneuromatosis was discovered incidentally during surgery for a retroperitoneal malignant peripheral nerve sheath tumor^[^13^]^. Similar to our case, visceral involvement was an unexpected intraoperative finding rather than the primary indication for surgery. However, that report involved ganglioneuromatosis in a younger patient undergoing oncologic surgery, whereas our patient had diffuse visceral neurofibromatous lesions encountered during biliary reconstruction for choledocholithiasis.

To better contextualize the present case, we have summarized previously reported cases of intra-abdominal neurofibromatosis in Table 1. Although gastrointestinal involvement in NF1 has been described, diffuse visceral neurofibromas encountered during biliary reconstruction remain exceedingly rare.

**Table 1:** Summary of some previously reported cases of intra-abdominal neurofibromatosis with gastrointestinal involvement.

REF	Author (year)	Age/sex	NF1 status	Clinical presentation	Site of involvement	Management	Outcome
10	Fu *et al* (2026)	17/F	NF1	Right lower quadrant pain	Small bowel mesentery	Appendectomy	Favorable
5	Ait Ali *et al* (2021)	84/M	NF1	Acute intestinal obstruction intussusception	Small bowel	Segmental resection	Favorable
14	Gandhi *et al* (2019)	68/M	NF1	Rectal bleeding with intermittent melena	Jejunum	Segmental resection	Favorable
11	Al-Harake *et al* (2013)	48/F	No associated Syndrome	Acute intestinal obstruction intussusception	Ileum	Segmental resection	Favorable
12	Fujisawa *et al* (2011)	26/M	NF1	Incidentally discovered	Periportal, mesentery and GI tract	Cholecystectomy	Favorable
13	Thway *et al* (2009)	32/M	NF1	Recurrent small bowel obstruction	Multiple intra-abdominal and retroperitoneal	Segmental resection	Favorable

NF1, Neurofibromatosis type 1; ERCP, endoscopic retrograde cholangiopancreatography; RUQ, right upper quadrant; CBD, common bile duct; CHD, common hepatic duct; MRCP, magnetic resonance cholangiopancreatography; EF, ejection fraction; PO, per oral.

In contrast to the skin lesions seen in NF1, gastrointestinal involvement is often asymptomatic and may remain undetected for years^[^14,15^]^. In the present case, the patient did not report any gastrointestinal symptoms suggestive of visceral neurofibromatosis, such as obstruction, gastrointestinal bleeding, or an abdominal mass. Furthermore, the previous cholecystectomy had been performed 8 years earlier, and biliary reconstruction or gastrointestinal anastomosis is not required during laparoscopic cholecystectomy. Therefore, the visceral lesions may not have posed a technical challenge or attracted particular surgical attention at that time. In addition, the medical records from the previous operation were not available, nor did the patient report any prior intraoperative findings suggestive of gastrointestinal involvement. Consequently, the visceral neurofibromas were identified only during the present surgical exploration. Additionally, patients with gastrointestinal NF1 may require intra-abdominal surgical interventions, such as choledochotomy. The presence of neurofibromas in the abdomen, especially in the small intestine, complicates and obscures the surgical field, making delicate interventions difficult ^[^5^]^.

The treatment of choice for CBD stones is ERCP), which allows physicians to explore and clear stones from the CBD. In patients with multiple large stones, ERCP is not feasible, and open surgical techniques are more effective, as in our case^[^16–18^]^.

This case emphasizes the challenges encountered during surgery on a patient with intra-abdominal neurofibroma, which made the operative process difficult, especially due to a combination of adhesions from prior surgeries. Another consideration for the surgeon was obstruction of the lumen, as a consequence of growth or thickening of neurofibromatosis lesions at the anastomosis site. The patient’s preferred method of treatment was choledochotomy. This surgical intervention required careful planning with a delicate anastomosis, which was important to minimize complications, such as bile leakage postoperatively^[^16^]^.

Histopathological confirmation of the visceral lesions was not obtained, as the lesions were not biopsied during surgery. The intraoperative diagnosis was based on their characteristic gross appearance in a patient with established NF1. We acknowledge the lack of histopathological confirmation as a limitation of this case report.

The presence of neurofibroma lesions posed difficulties by obstructing the surgical site and made it difficult for the surgeon to find appropriate space for anastomosis, particularly in identifying a region of the small bowel without neurofibroma involvement. An additional factor that the surgeon had to take note of was the possibility of growth of these lesions at the anastomosis site, which could consequently cause intestinal obstruction.

## Conclusions

Patients with generalized skin lesions of NF1 may require surgical intervention in the abdomen and gastrointestinal tract. Visceral lesions are rare but may significantly complicate abdominal surgery. Surgeons should be aware of possible diffuse visceral lesions, which can make identification of a suitable, lesion-free segment for anastomosis challenging. Careful intraoperative assessment is essential to ensure safe reconstruction and avoid postoperative complications.

## Data Availability

The datasets used during the current study are available from the corresponding author on reasonable request.
